# A preliminary study evaluating self-reported effects of cannabis and cannabinoids on neuropathic pain and pain medication use in people with spinal cord injury

**DOI:** 10.3389/fpain.2023.1297223

**Published:** 2023-12-21

**Authors:** Kristiina Kinnunen, Linda E. Robayo, Nicholas P. Cherup, Scott I. Frank, Eva Widerström-Noga

**Affiliations:** ^1^ The Miami Project to Cure Paralysis, University of Miami, Miami, FL, United States; ^2^ Neuroscience Graduate Program, University of Miami, Miami, FL, United States; ^3^ Department of Neurological Surgery, University of Miami, Miami, FL, United States

**Keywords:** cannabis, cannabinoids, neuropathic pain, pain medication, spinal cord injury

## Abstract

Approximately 60% of individuals with a spinal cord injury (SCI) experience neuropathic pain, which often persists despite the use of various pharmacological treatments. Increasingly, the potential analgesic effects of cannabis and cannabinoid products have been studied; however, little research has been conducted among those with SCI-related neuropathic pain. Therefore, the primary objective of the study was to investigate the perceived effects of cannabis and cannabinoid use on neuropathic pain among those who were currently or had previously used these approaches. Additionally, the study aimed to determine if common pain medications are being substituted by cannabis and cannabinoids. Participants (*N* = 342) were recruited from existing opt-in listserv sources within the United States. Of those, 227 met the inclusion criteria and were enrolled in the study. The participants took part in an anonymous online survey regarding past and current use of cannabis and their perceived effects on neuropathic pain, including the use of pain medication. Those in the sample reported average neuropathic pain intensity scores over the past week of 6.8 ± 2.1 (0 to 10 scale), reflecting a high moderate to severe level of pain. Additionally, 87.9% noted that cannabis reduced their neuropathic pain intensity by more than 30%, and 92.3% reported that cannabis helped them to better deal with their neuropathic pain symptoms. Most participants (83.3%) also reported substituting their pain medications with cannabis, with the most substituted medication categories being opioids (47.0%), gabapentinoids (42.8%) and over-the-counter pain medications (42.2%). These preliminary results suggest that cannabis and cannabinoids may be effective in reducing neuropathic pain among those with SCI and may help to limit the need for certain pain medications.

## 1. Introduction

Spinal cord injury (SCI)-associated chronic pain has an estimated prevalence of 68% (1) and has been described as one of the most challenging problems to manage in people living with SCI (2). Translational research findings have helped clinicians to develop a nuanced understanding of how pain emerges and persists in the body, with SCI-related chronic pain commonly being classified as nociceptive or neuropathic (1, 3, 4). Notably, neuropathic pain is defined as a type of pain that arises as a consequence of an injury or disease affecting the somatosensory nervous system (5) and is understood to be one of the most severe and treatment-resistant types of pain in this population (6). While such symptoms are predominately treated with prescription medications such as anticonvulsants, antidepressants, opioids, or local anesthetics (7), each approach carries undesirable side effects (8). Research also suggests that pharmacological treatment is often inadequate resulting in only a 20–30% reduction in pain intensity in a fraction of patients (9). Given these data, exploring alternative treatment options to reduce or manage neuropathic pain in individuals with SCI is critical not only to decreasing the amount of suffering experienced but also for enhancing their quality of life (QoL) (10).

One treatment approach that may be a potential alternative is cannabis. The cannabis plant is a source of several compounds known as cannabinoids. The Δ^9^-tetrahydrocannabinol (THC) and cannabidiol (CBD) are two of these compounds that have been extensively investigated in preclinical and clinical studies of neuropathic pain [See Reviews (11, 12)]. The administration of CBD and THC in animal models has demonstrated significant analgesic effects by reducing pain-related behaviors (13), including allodynia (5, 14). It is known that CBD and THC interact with two G-protein-coupled receptors, the cannabinoid receptor type 1 (CB_1_R) and the cannabinoid receptor type 2 (CB_2_R). Specifically, THC is a CB_1_R and CB_2_R partial agonist whereas CBD is an antagonist of CB_1_R/CB_2_R receptor agonists (15). Interestingly, activation of CB_1_R/CB_2_R receptors can suppress nociceptive transmission (16), and because CB_2_R is expressed at elevated levels in the CNS, it has been considered a therapeutic target for the treatment of neuropathic pain (17). Activation of these receptors may be responsible for their antinociceptive effects, in part, by reducing the neuronal release of excitatory neurotransmitters including glutamate (18). However, the overall analgesic properties of cannabis/cannabinoids likely include multiple cellular and molecular mechanisms such as inhibition of neurotransmitter release, modulation of neuronal excitability and a reduction in neuroinflammation [See Review (19)].

The cannabis plant and cannabinoids have been increasingly studied as potential therapeutic agents for several health-related conditions, including chronic pain (10, 11, 20, 21). Regular cannabis use among people with SCI has been associated with improved sleep, decreased depression, and anxiety, as well as improved social interaction, which together contribute to better QoL (10, 22–26). While the analgesic effects of the cannabis plant have been supported with anecdotal evidence, research is beginning to support its use. For example, Andersen et al. (22) examined both recreational and medical use of cannabis in 537 people with traumatic SCI and found that cannabis use was more prevalent among those with SCI compared to the general population (22). Among cannabis users with SCI, 59% reported a positive effect of cannabis on pain and spasticity, and 4% for other medical reasons such as depression and anxiety. Similarly, a recent meta-analysis presented that cannabis users experienced a 30% greater reduction in chronic pain compared to the placebo, along with improvements in symptoms of spasticity (27). In the United States, a study (*N* = 244) regarding the therapeutic use of cannabis showed that 22.5% of the participants reported using cannabis at least once a month for pain relief (70.4%), and/or spasticity (46.3%) (23). Individuals with SCI also report cannabis use for pain because prescribed medications are often found to be ineffective or have side effects that they do not otherwise experience with cannabis (10). Moreover, a qualitative, single-focus group study in the United States concluded that cannabis use allowed some individuals with SCI to discontinue their prescribed anxiety- and narcotic medications (24). Similar findings were also confirmed by Bourke and colleagues (10), who showed that individuals with SCI reported choosing to use cannabis for pain management over traditionally prescribed pain medications (10). Such findings are crucial as the opioid epidemic continues to be a major health concern across the United States population (28).

While cannabis-related research has shown promising results in reducing pain and improving the overall QoL in those with SCI, more research is necessary to better understand its effects specifically on neuropathic pain and pain medication use within this population. A recent survey study also explored attitudes toward the medical use of cannabis and its usage in people with SCI (29). The survey had four primary focuses; (1) to examine attitudes towards the medical use of cannabis, (2) to determine what the experiences of using cannabis were like, (3) what the perceptions of the benefits and risks regarding cannabis, and (4) the extent of knowledge of both medical and recreational cannabis use. Among the 353 responses from 39 states of the United States, 30.3% reported using medical cannabis, with 11.8% describing past use, and 36.6% indicating no prior use. Reasons for cannabis use included pain, insomnia, and anxiety relief, hope for reduced reliance on medications, and recommendations from doctors and friends (29). Unfortunately, this study lacked details on dosage, type of cannabinoids consumed (CBD, THC), pain subtypes (nociceptive and/or neuropathic), pain intensity, and differentiation between acute and chronic pain. In addition, a recent study found no significant effect of THC and CBD compared to placebo on neuropathic pain intensity (30). Thus, the main purpose of the present study was to investigate the self-reported effects of cannabis and cannabinoids on (1) neuropathic pain unpleasantness and interference with sleep, mood, and daily activities, in addition to pain intensity, as well as (2) pain medication use in people with SCI. Furthermore, we assessed the type of cannabinoids consumed, frequency of consumption, dosage and perceived side effects, as well as the preferred method of use, timing and the strains used.

## 2. Methods

### 2.1. Study design

The study included an anonymous online survey created with Qualtrics.com (Supplementary Material), regarding cannabis and cannabinoid use in people experiencing neuropathic pain after their SCI. The authors reviewed previous research and surveys conducted concerning cannabis/cannabinoids and pain (23, 29, 31–33) and created a 45-item survey that focused specifically on SCI-associated neuropathic pain, as well as the effects of cannabis/cannabinoids on the demand for pain medication. Prior to starting the survey, participants were provided with a brief description of cannabis/cannabinoids and how pain is categorized (nociceptive/neuropathic), in addition to an informed consent statement. After agreeing to continue with the survey, participants were asked about their age, injury, and pain symptoms to confirm that they met the inclusion criteria described in Section 2.3; Participant Recruitment.

### 2.2. Survey questions

#### 2.2.1. Demographics

The survey incorporated demographic questions including age, gender identity, ethnicity, education level, demographic region, marital status, housing arrangement and employment status.

#### 2.2.2. Injury and neuropathic pain characteristics

To assess information regarding participants' SCI characteristics, they were asked questions regarding the level (cervical, thoracic, lumbar, other), cause of injury (fall, motor vehicle accident, violence, sports, other), and the grade of the injury (complete, incomplete), as well as time since the SCI.

Information regarding SCI-related neuropathic pain was assessed through questions measured on a 10-point Likert scale, including pain intensity (0 indicating no pain, 10 indicating most intense pain imaginable), unpleasantness (0 indicating no unpleasantness, 10 indicating most unpleasant pain imaginable), ability to deal with the pain (0 indicating not hard at all, 10 indicating extremely hard), as well as pain interference (0 indicating no interference, 10 indicating extreme interference) with mood, sleep, and daily activities (34).

Participants' usage of pain medication was evaluated by inquiring about their current use of several categories of pain medications, including Gabapentinoids (Gabapentin, Pregabalin), Serotonin and Norepinephrine Reuptake Inhibitors SSRIs (Duloxetine, Venlafaxine), Tricyclic Antidepressants (Nortriptyline, Amitriptyline), Topical analgesics (5% Lidocaine, 8% Capsaicin), Opioids (Tramadol, Tapentadol, Morphine, Oxycodone), Neurotoxin (Botulinum toxin), and Over-The-Counter Pain Medications [acetaminophen, aspirin, and nonsteroidal anti-inflammatory drugs (NSAIDs) such as ibuprofen, naproxen, and diclofenac]. To assist participants who might not be familiar with the medication categories, but rather the brand names of medications, a list of the most prescribed brand names of each category was provided prior to their selection.

#### 2.2.3. Cannabis/cannabinoid use

All participants were asked about their history of cannabis and/or cannabinoid use, including past use, current use, and whether they used cannabis and cannabinoids before and/or after the onset of their SCI. The patterns of cannabis and cannabinoid use were examined through questions regarding the amount used weekly (measured in grams), the specific cannabinoids used (THC + CBD, CBD only, or other), the method of consumption (e.g., Flower, oil, vaporize, edible), the timing of use (morning, evening, both, no preference), as well as the frequency of use (multiple times a day, daily, weekly, monthly, less than monthly). Participants who indicated THC use were further asked about the strain used (Indica, Sativa, Hybrid, Unsure, No preference).

To assess whether cannabis and cannabinoids were being utilized as alternatives to pain medications, participants were asked whether they had or were currently substituting pain medications with cannabis and/or cannabinoids. Participants who responded substituting pain medications with cannabis, were provided an opportunity to specify the medication they replaced with cannabis.

Participants were also asked about the effects of cannabis and cannabinoids on several aspects of their neuropathic pain, including pain intensity, pain unpleasantness, and interference with sleep, mood, and activities, as well as their ability to deal with their pain. Furthermore, they were asked if cannabis/cannabinoids had any impact on other important factors in their lives, such as stress, depression/anxiety, appetite, spasticity, insomnia, focus/concentration, and relaxation. Participants who reported cannabis/cannabinoids leading to reduced neuropathic pain intensity were further asked to indicate the percentage by which it/they reduced their pain (less than 25%, 30–50%, 51–75%, more than 75%). Additionally, participants were asked to rate how much cannabis helped them to deal with their neuropathic pain (ranging from “Not at all” to “A lot”), as well as the overall effect of cannabis and/or cannabinoids on their global wellbeing (“Very much improved”, “Much improved”, “Minimally improved”, “No change”, “Minimally worse”, “Much worse”, “Very much worse”). Finally, a question regarding any negative side effects experienced from cannabis was also included in the questionnaire.

### 2.3. Participant recruitment

This study was approved by the University of Miami Institutional Review Board (IRB). Participants were recruited via email from an existing opt-in listserv of the Miami Project to Cure Paralysis, The South Florida SCI Model Systems listserv, the North American SCI Consortium, and local SCI support groups (Broward, Pam-Beach, and Miami-Dade). Flyers were posted throughout the University of Miami. Inclusion criteria required participants to identify themselves as individuals who have a history of cannabis and/or cannabinoid use, be over the age of 18, and have experienced SCI-related neuropathic pain for at least three months. All participants resided in the United States and were able to communicate in English. Individuals who did not meet the inclusion criteria were excluded from the study.

### 2.4. Data collection

All survey responses were self-reported through an anonymous survey. Given the partial illegal status of cannabis use in some states of the United States, it was crucial to prioritize confidentiality and anonymity and ensure that no identifiable information was obtained throughout the study. The survey was open for 6 full weeks, and 314 individuals accessed the survey and consented to participate in the study.

### 2.5. Data analysis

Data summaries and results were obtained using statistical software GraphPad Prism v9, Python and generated reports from Qualtrics.com. Nominal data were summarized into frequencies and percentages, while data in the interval- and ratio scales were expressed in means and standard deviations. To investigate associations between neuropathic pain intensity, unpleasantness, and pain interference with activities, mood, and sleep, we calculated Pearson correlation coefficients.

## 3. Results

### 3.1. Demographic characteristics

**Figure 1:**
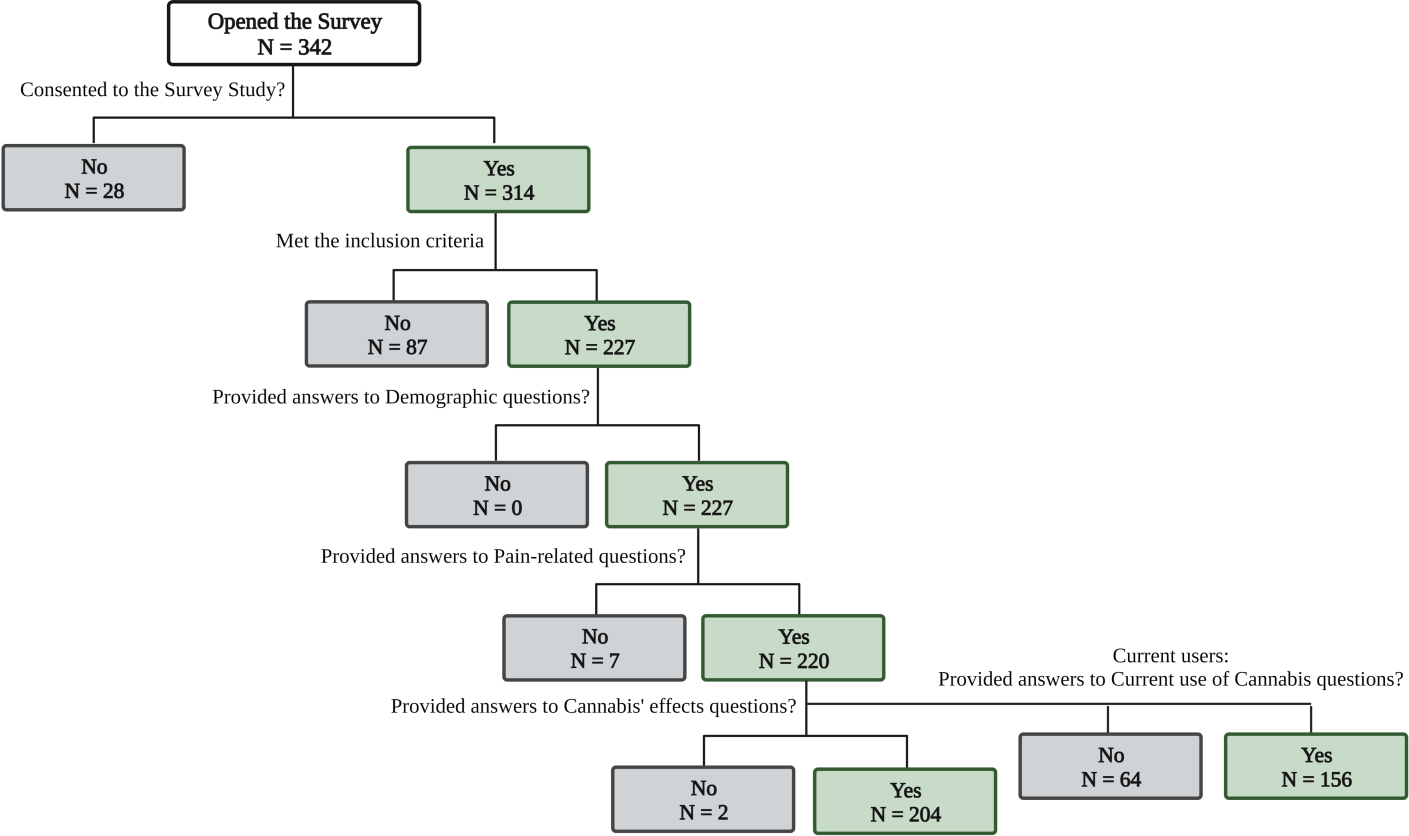
Flowchart of participants’ responses collected.

A total of 342 participants within the United States opened the survey, with 227 of them meeting the inclusion criteria (See Figure 1).

All demographic information is summarized in Table 1. Participants had previously experienced an SCI and were currently suffering from neuropathic pain. Additionally, they had either current- or past experience with cannabis and/or cannabinoid use. Among all participants, 67.3% identified as males, 31.4% as females, and 1.3% as other or preferred not to answer. Forty-one percent of the participants were between 31 and 45 years of age, and most identified as Caucasian (73.1%). Most participants either lived with someone (67.8%), or lived alone (28.6%), with 43.6% of the sample reporting being single or never married, 35.7% married, or in a domestic relationship, and 15.9% divorced. Among the participants, 30.8% were unable to work, 22.9% were unemployed, and 13.7% were either employed part-time or full-time (14.1%).

**Table 1:** Demographics.

Demographic characteristics (*n* = 227)	
*n* (%)	
Age range	
18–30	28 (12.3%)
31–45	93 (41.0%)
46–60	79 (34.8%)
61 or older	27 (11.9%)
Gender^a^	
Male	152 (67.3%)
Female	71 (31.4%)
Transgender male	1 (0.4%)
Transgender female	0 (0.0%)
Gender variant/non-conforming	0 (0.0%)
Prefer not to answer	2 (0.9%)
Ethnicity^b^	
White	166 (73.1%)
Black or African-American	32 (14.1%)
American Indian or Alaskan Native	7 (3.1%)
Asian	4 (1.8%)
Prefer not to answer	12 (5.3%)
Other	16 (7.0%)
Hispanic, Latino or Spanish origin	
Yes	34 (15.0%)
No	183 (80.6%)
Prefer not to answer	10 (4.4%)
Housing arrangement	
Living with someone	154 (67.8%)
Living alone	65 (28.6%)
Nursing home/facility	2 (0.9%)
Homeless	1 (0.4%)
Other	5 (2.2%)
Education	
Less than a high school diploma	13 (5.7%)
High school degree or equivalent	37 (16.3%)
Some college, no degree	62 (27.3%)
Associate degree	33 (14.5%)
Bachelor's degree	52 (22.9%)
Master's degree	20 (8.8%)
Doctorate or professional degree	10 (4.4%)
Marital status	
Single	99 (43.6%)
Married or in a domestic relationship	81 (35.7%)
Widower	6 (2.6%)
Divorced	36 (15.9%)
Separated	5 (2.2%)
Employment^b^	
Employed full time	32 (14.1%)
Employed part-time	31 (13.7%)
Unemployed	52 (22.9%)
Student	8 (3.5%)
Retired	33 (14.5%)
Self-employed	23 (10.1%)
Unable to work	70 (30.8%)

^a^ One participant did not respond to this question.

^b^ Participants were able to select multiple answers.

### 3.2. Injury and neuropathic pain characteristics

Injury and neuropathic pain characteristics are summarized in Table 2. Regarding the SCI-related characteristics, 43.8% of the participants had experienced a cervical level injury, 45.1% a thoracic level injury and 13.3% a lumbar level injury. Among all participants, 58.8% had experienced an incomplete SCI, while 37.6% reported a complete SCI. Most participants (54.4%) had been living with a spinal cord injury for 10 years or longer. The most common cause of SCI was motor vehicle accidents (44.7%), including pedestrian and non-pedestrian-related events.

**Table 2:** Injury and pain characteristics.

Injury characteristics (*n* = 226)^a^	
*n* (%)	
Level of SCI^b^	
Cervical	99 (43.8%)
Thoracic	102 (45.1%)
Lumbar	30 (13.3%)
Unsure	14 (6.2%)
Grade of SCI	
Complete	85 (37.6%)
Incomplete	133 (58.8%)
Unsure	8 (13.5%)
Time since SCI	
6–12 months	6 (2.7%)
1–3 years	18 (8.0%)
4–6 years	42 (18.6%)
7–9 years	37 (16.4%)
10 or more years	123 (54.4%)
Cause(s) of SCI^b^	
Motor vehicle accident—pedestrian	25 (11.1%)
Motor vehicle accident—non-pedestrian	76 (33.6%)
Violence	23 (10.2%)
Fall	34 (15.0%)
Sport-related injury or Other	80 (35.4%)
Pain characteristics (*n* = 220)	
Mean (SD)	
Pain intensity	6.8 (2.1)
Pain unpleasantness	6.9 (2.2)
Hard to deal with pain	5.6 (2.7)
Pain interference with sleep	6.2 (3.0)
Pain interference with mood	5.9 (2.7)
Pain interference with daily activities	5.8 (2.8)

^a^ One participant did not respond to these questions.

^b^ Participants were able to select multiple answers.

The average neuropathic pain intensity was reported as 6.8 ± 2.1 out of 10, with a similar average for pain unpleasantness (6.9 ± 2.2). Participants also reported an average difficulty level of 5.6 ± 2.7 when dealing with their neuropathic pain. In terms of the interference caused by neuropathic pain, participants reported an average score of 6.2 ± 3.0 out of 10 for its impact on sleep, 5.9 ± 2.7 on mood, and 5.8 ± 2.8 on daily activities.

### 3.3. Use of cannabis/cannabinoids

**Figure 2:**
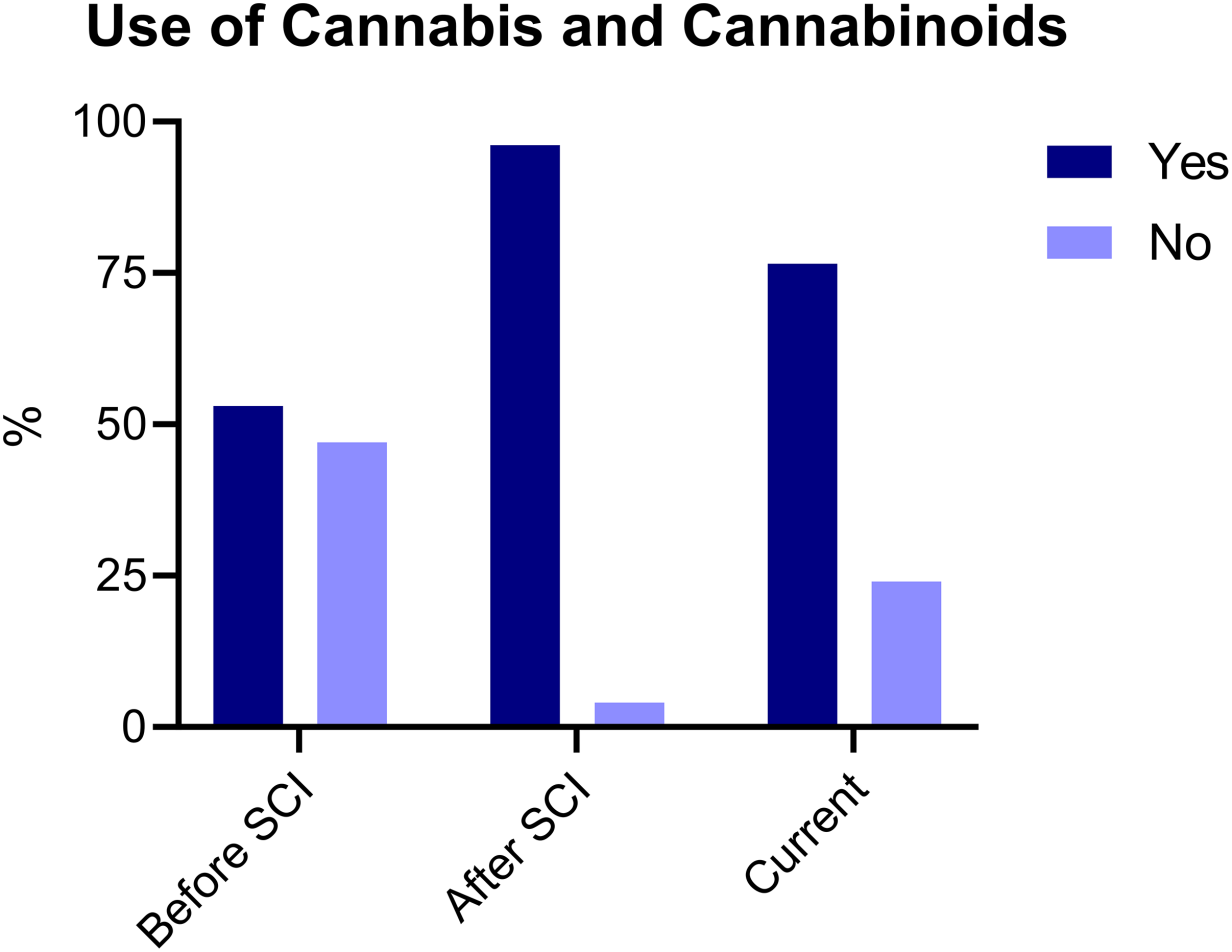
Use of cannabis and cannabinoids before SCI, after SCI and current. Participants responded “yes” or “no” for each condition (before, after and current). Responses are reported as percentages (%).

Out of 204 participants who had ever used cannabis, 52.9% had used cannabis or cannabinoids before the occurrence of their SCI, and 96.1% after the onset of their SCI. A significant majority of these participants, comprising 76.5%, reported currently using cannabis or cannabinoids (See Figure 2). Information regarding participants' current cannabis and cannabinoid use is summarized in Table 3. Among the current users of cannabis and cannabinoids, most respondents reported using cannabis products once (33.3%) or twice per day (28.3%). The most frequently reported amount used on a weekly basis was between 2 and 4 grams (23.7%). Alternatively, the most reported methods of consumption were edibles (53.8%), vaporization (39.7%) and joints/blunts (36.5%). Additionally, respondents consumed cannabis or cannabis-based products containing THC (84.0%) and CBD (51.3%) only or in combination with THC. With respect to THC-containing cannabis strains, the most predominant strains used were Indica (53.8%) and Hybrid (51.9%).

**Table 3:** Characteristics of cannabis and cannabinoids current use.

Characteristics of cannabis/cannabinoids current use (*n* = 156)	
*n* (%)	
Frequency of use	
Less than monthly	6 (3.8%)
Monthly	9 (5.8%)
Weekly	18 (11.5%)
Daily or almost daily	62 (39.7%)
Multiple times a day	61 (39.1%)
Daily frequency of use	
Once a day	52 (33.3%)
Twice a day	41 (28.3%)
Three times a day	27 (17.3%)
Four or more times a day	36 (23.1%)
Time of the day of use^b^	
Morning	39 (25.0%)
During the day	32 (20.5%)
Evening	106 (67.9%)
No preference	46 (29.5%)
Modality^b^	
Pipe	39 (25.0%)
Joint/Blunt	57 (36.5%)
Bong	26 (16.7%)
Vaporize	62 (39.7%)
Oil/Tincture	31 (19.9%)
Edible/Capsule/Food	84 (53.8%)
Topical (cream, lotion)	20 (12.8%)
Fresh Juice	4 (2.6%)
Other	5 (3.2%)
Composition^b^	
Cannabidiol (CBD)	80 (51.3%)
Delta-9-tetrahydrocannabinol (THC)	131 (84.0%)
I don’t know	11 (7.1%)
Other	9 (5.8%)
Amount used per week	
1 gram or less	25 (16.0%)
2–4 grams	37 (23.7%)
5–7 grams	28 (17.9%)
8–10 grams	18 (11.5%)
More than 10 grams	22 (14.1%)
I don’t know	19 (12.2%)
Other	7 (4.5%)
THC strain used^a,b^	
Indica	84 (53.8%)
Sativa	65 (41.7%)
Hybrid	81 (51.9%)
Not sure	9 (5.8%)

^a^ Answered by current users of THC only (*n* = 131).

^b^ Participants were able to select multiple answers.

### 3.4. Effects of cannabis/cannabinoids

**Figure 3:**
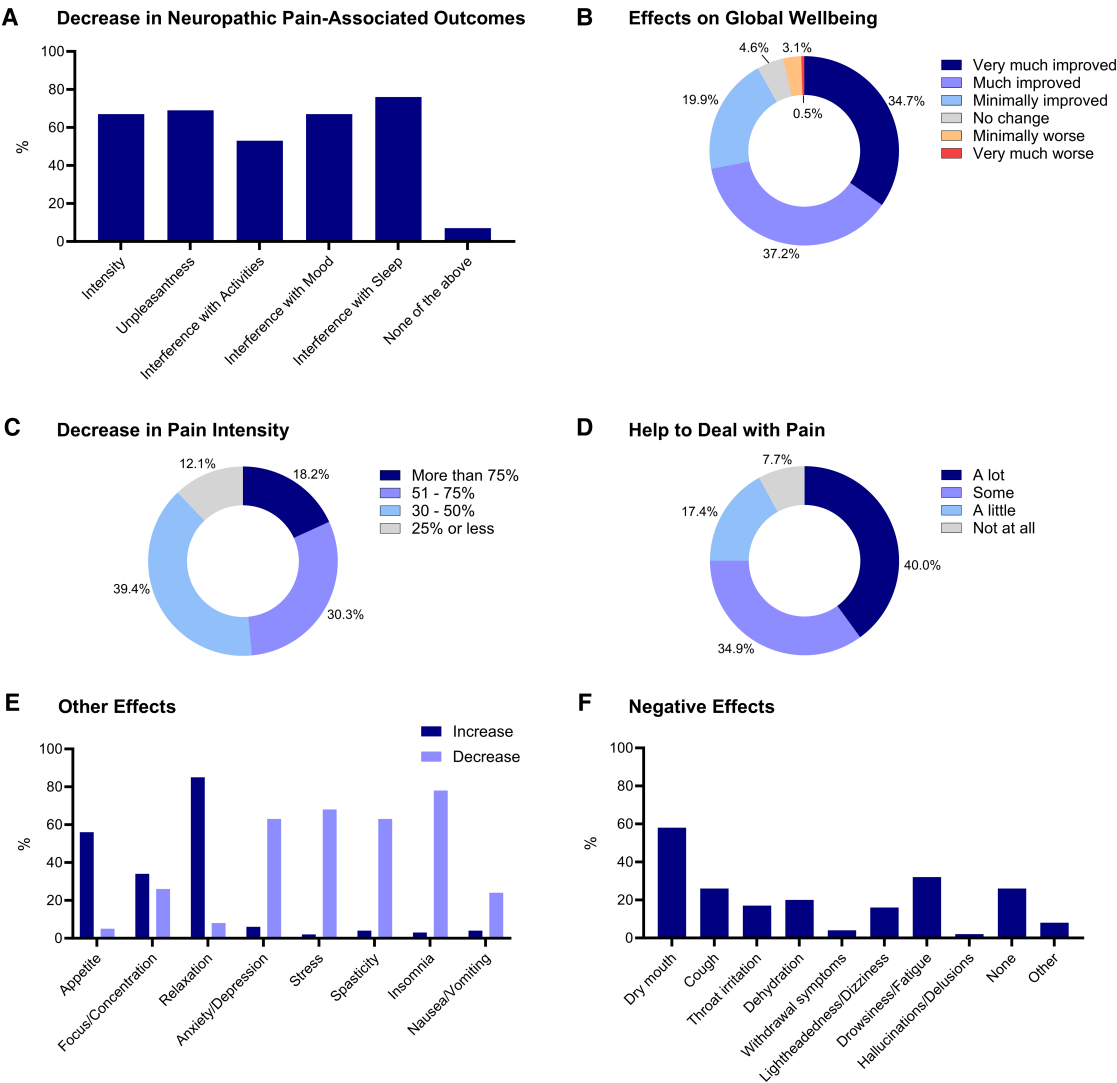
Decrease in neuropathic pain associated outcomes (**A**), effects on global well-being (**B**), decrease in pain intensity (**C**), help to deal with pain (**D**) other effects (**E**) and negative effects (**F**) associated with Cannabis and Cannabinoids’ use. Responses are reported as percentages (%).

A substantial majority of the participants with a history of cannabis/cannabinoid use, accounting for 93.5% of the sample (*n* = 186), reported experiencing at least some benefit from cannabis, either by decreasing neuropathic pain intensity, unpleasantness or interference with activities, mood, and sleep (See Figure 3A). Overall, 71.9% of participants reported cannabis and cannabinoids improving their global well-being “much” (*n* = 73) or “very much” (*n* = 68) (See Figure 3B). Among the participants who experienced reduced neuropathic pain intensity due to cannabis and cannabinoid use, 87.9% reported a decrease of more than 30% (*n* = 110), with 18.2% reporting more than a 75% reduction (*n* = 24) (See Figure 3C). Furthermore, 40.0% (*n* = 78) stated that cannabis and cannabinoids did help them deal with their neuropathic pain “a lot” (See Figure 3D). Additionally, participants reported the use of cannabis and cannabinoids decreased their stress (68.4%, *n* = 134), anxiety and depression (62.8%, *n* = 123), spasticity (62.8%, *n* = 123), insomnia (77.6%, *n* = 152), and feelings of nausea and vomiting (24.5%, *n* = 48) while increasing their relaxation (85.2%, *n* = 167), as well as appetite (56.1%, *n* = 110) and focus and concentration (33.7%, *n* = 66) (See Figure 3E). Negative effects from cannabis and cannabinoids were reported mostly as dry mouth (58.2%, *n* = 114), drowsiness (31.6%, *n* = 62), and cough (26.0%, *n* = 51) (See Figure 3F). In addition, (25.5%, *n* = 50) of the participants reported experiencing no negative side effects from cannabis and cannabinoids.

**Figure 4:**
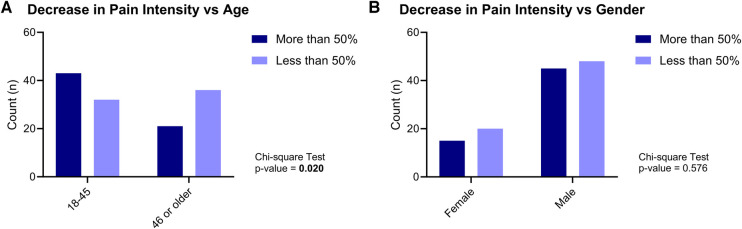
Decrease in neuropathic pain intensity vs. age (**A**) and gender (**B**).

In addition, we conducted subgroup analyses to investigate differences in reported decrease in neuropathic pain intensity associated with cannabis and cannabinoids use, across age and gender. The sample was dichotomized into “18–45” (*n* = 121) and “46 or older” (*n* = 106) to provide subgroups relatively similar in size. Similarly, the sample was also dichotomized into “less than 50%” (*n* = 68) and “more than 50%” (*n* = 64) categories for the decrease in pain intensity variable to allow for comparison. For gender, only males (*n* = 152) and females (*n* = 71) were compared because of the small sample sizes of the other categories (See Table 1). Chi-square tests were performed and *p*-values of less than 0.05 were considered significant. No significant differences were found across gender (*p* = 0.576), with respect to decrease in pain intensity (See Figure 4A). However, there was a significant difference across age (*p* = 0.020) subgroups (See Figure 4B). This result indicates that among the participants who experienced reduced neuropathic pain intensity due to cannabis and cannabinoid use, a lower proportion of individuals 46 or older reported a decrease in pain intensity of more than 50%, compared to 18–45-year-old individuals. These results should be confirmed in larger studies and additional subgroup comparisons should be also explored.

### 3.5. Current medication use and substitution with cannabis/cannabinoids

**Figure 5:**
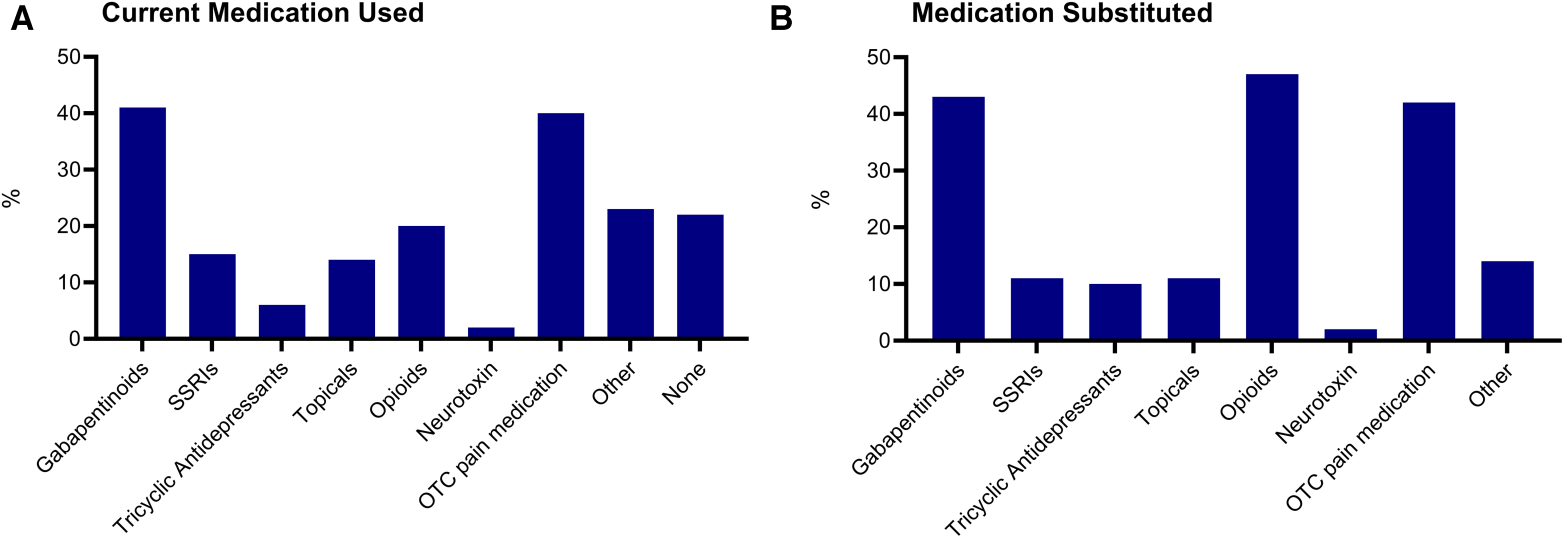
Medication use and substitution. Medication categories currently used (**A**) and currently substituted with cannabis and cannabinoids (**B**) SSRIs, selective serotonin reuptake inhibitor. OTC, over-the-counter.

The most common currently used medication categories were gabapentinoids (40.9%, *n* = 90), over-the-counter pain medications (40.0%, *n* = 88), and opioids (20.5%, *n* = 45) (See Figure 5A). Additionally, 83.3% of participants had used cannabis/cannabinoids to substitute their pain medication (*n* = 170). The results indicated that the most substituted medications were opioids (47.0%, *n* = 78), gabapentinoids (42.8%, *n* = 71) and over-the-counter pain medications (42.2%, *n* = 70) (See Figure 5B).

### 3.6. Associations among neuropathic pain characteristics

**Figure 6:**
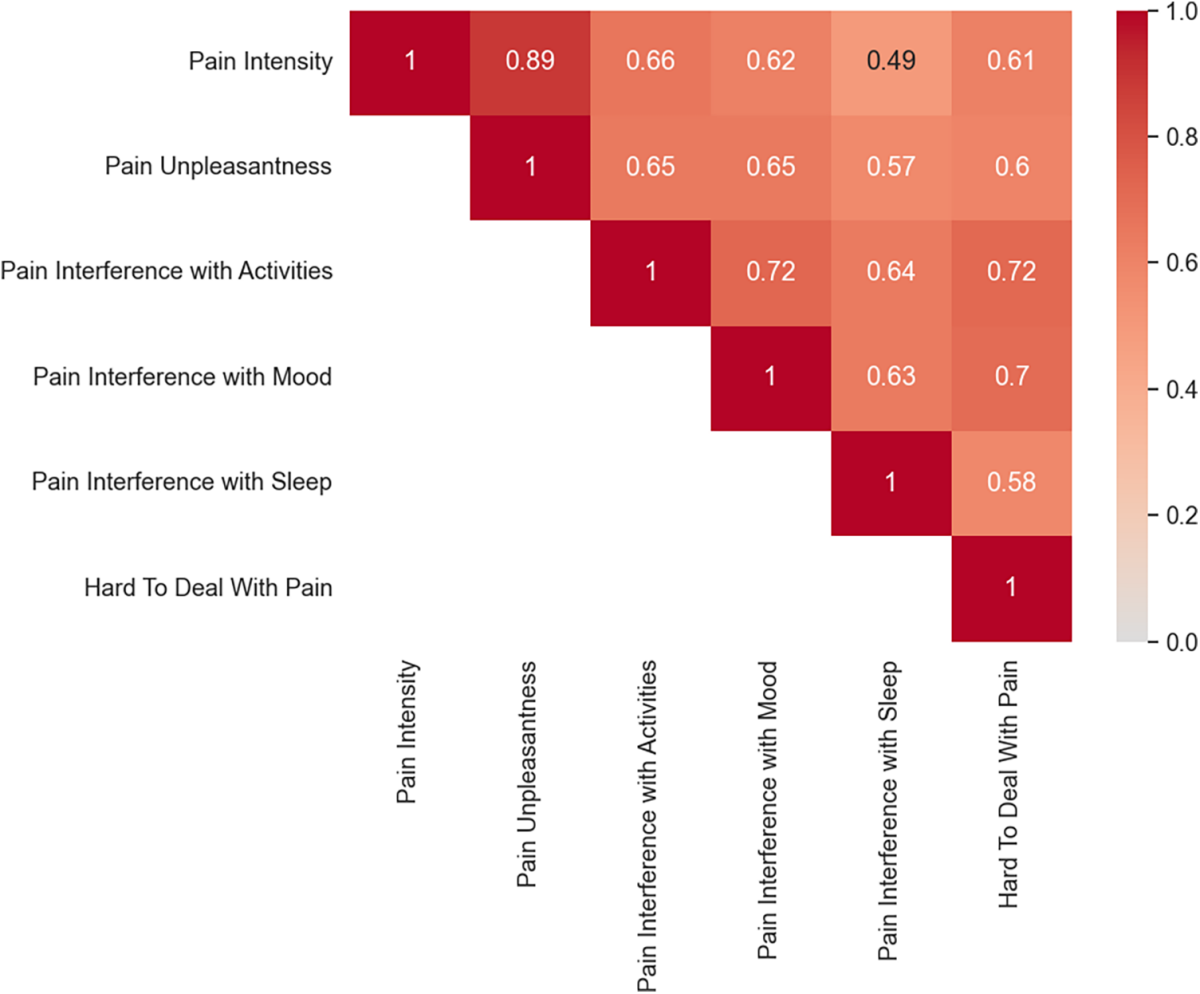
Correlation matrix for neuropathic pain characteristics. Darker red colors reflect greater positive correlations compared to lighter red.

Pearson correlations indicated that pain intensity ratings were positively and highly correlated with pain unpleasantness (*r* = 0.89, *p* = <0.001), moderately correlated with pain interference with activities (*r* = 0.66, *p* = <0.001), mood (*r* = 0.62, *p* = <0.001), and dealing with pain (*r* = 0.61, *p* = <0.001), and weakly correlated with pain interference with sleep (*r* = 0.49, *p* = <0.001) (See Figure 6).

## 4. Discussion

The current study is one of the first cross-sectional survey studies specifically focused on the perceived effects of cannabis and cannabinoids on neuropathic pain and pain medication use in people with SCI. Our findings indicate that individuals using cannabis and/or cannabinoids experience a reduction in neuropathic pain intensity, pain unpleasantness, pain interference and other pain-associated factors (e.g., stress, anxiety/depression, insomnia). In addition, our results suggest that many participants are voluntarily substituting their pain medication with cannabis and/or cannabinoids. These findings support the evidence from previous studies (23, 29, 31–33), emphasizing the need for larger clinical trials evaluating the analgesic effects of cannabis and/or cannabinoids within this heterogeneous and often treatment-resistant population.

In the present study, 92.3% of current cannabis and/or cannabinoid users reported that cannabis/cannabinoids help them deal with their neuropathic pain. This is far greater than a 2017 study from Denmark, which indicated that only 56% of current cannabis users reported positive effects on pain and spasticity with cannabis use (22). Observed disparities between these two studies may be the result of different dosing of cannabis, as well as the concentration of different cannabinoids (CBD/THC) in the formulations consumed. It may also be the case that survey respondents in the current sample had greater accessibility to cannabis/cannabinoid products as the United States is increasingly approving the use of cannabis for medical purposes. Our sample data focused on neuropathic pain also suggests that pain intensity scores were rated as slightly higher (6.8 ± 2.1) than those observed by previous authors (5.1 ± 2.3 and 5.3 ± 2.3) (35, 36). Given these findings, it might be that those with severe neuropathic pain symptoms may be using cannabis/cannabinoids as a primary pain management strategy over other commonly prescribed medications.

Participants also reported high pain interference mean scores across domains of sleep (6.2 ± 3.0), mood (5.9 ± 2.7), and daily activities (5.8 ± 2.8). Previous research suggests that pain is one of the most commonly reported symptoms interfering with sleep and daily activities among individuals with SCI (37). Emotional distress has also been associated with neuropathic pain to a greater extent than the severity of the SCI, *per se* (e.g., time since injury, level, and completeness) (38). Similarly, spasticity may be exacerbated by neuropathic pain symptoms post-SCI (39). Thus, a reduction in any one of these two comorbidities would conceivably improve QoL among individuals living with this life-altering condition. Subsequently, cannabis has shown great medicinal benefits for many chronic pain symptoms including social and emotional disability scores and measures of sleep quality (40). The ability to control one's emotions or feel better about oneself and the ability to achieve better sleep throughout the night likely plays a large role in improving overall QoL. While the aforementioned study did include some SCI participants, a separate survey in Colorado confirmed their findings and reported a significant number of individuals with SCI used cannabis to reduce spasticity and improve their quality of sleep (24).

When examining the use of cannabis and/or cannabinoids pre- and post-SCI, 52.9% of participants reported using cannabis and/or cannabinoids before their injury occurred, while 96.1% reported use of cannabis or cannabinoids after their injury. These results indicate that despite that a greater proportion of people reported using cannabis before their SCI, a sizable proportion began using it after their injury. This trend suggests that individuals are seeking the potential benefits of cannabis and cannabinoids to alleviate their SCI-related symptoms. Moreover, these findings contradict previous research indicating that those with SCI and traumatic brain injury (TBI) reported a decrease in their cannabis use post-injury (24). These results might be due to increased use of other medications that might be interacting with cannabis and cannabinoids, as well as social stigma (24). A significant majority of the participants in the current sample (76.5%) also reported current use of cannabis. Considering that the average time since injury was 10 years or greater for most of the sample, cannabis may be viewed as a helpful therapeutic option to mitigate many symptoms in this population, including pain. While preliminary, such evidence suggests that cannabis is perceived as beneficial for managing SCI-related neuropathic pain. However, future research should continue to examine the potential beneficial effects of this approach in larger randomized controlled clinical trials.

In addition to the effects of cannabis and cannabinoid use on neuropathic pain symptoms, we also aimed to investigate other possible effects of these compounds across other pain and SCI-associated symptoms. As a result, our findings suggest that most of our participants reported that cannabis use helped them to decrease psychological and physical symptoms of stress, anxiety/depression, spasticity and insomnia, as well as increase relaxation and appetite. These results align with those of other research studies conducted in similar populations with many seeing improvements in mood and sleep quality (24). It is also important to note that the negative effects of cannabis surveyed, including dry mouth, drowsiness and cough, were considered acute effects of cannabis/cannabinoids. Previous surveys revealed similar results in terms of acute side effects but found even fewer of their participants experienced such symptoms. For example, only 27 of 51 SCI participants felt negative effects such as decreased motivation and fatigue (24). Notably, the potential long-term effects of cannabis use, such as structural alterations in the brain and central nervous system or inflammation of airways due to smoke ingestion were not surveyed in the current study. However, many methods used to detect these abnormalities are not considered standard of care and may therefore go undetected. It may also be that other confounding conditions are contributing to such outcomes rather than cannabis itself (41).

Limited research has also been conducted on the comparative effectiveness of cannabis and prescribed medication for pain management in those living with SCI-associated neuropathic pain. Medical and pharmacy claim data does, however, suggest that when compared to non-injured and demographically matched opioid users, those with SCI are significantly more likely to be using short-acting, low-dose, and/or long-acting high-dose opioid medications (42). Those with SCI were also more likely to be on a morphine equivalent dose of their opioid prescription which increases the risk for drug dependence and the likelihood for other adverse events. Therefore, as a secondary objective, we aimed to investigate whether individuals are substituting their prescribed pain medication with cannabis and/or cannabinoids. Previous findings from Drossel et al. (23) suggested that when compared to non-cannabis users, a greater percentage of those who were currently using cannabis were also taking other prescribed medications to treat pain, spasticity, and neuropathy. However, such differences were found to be nonsignificant (23). Conversely, Stillman et al., (29) compared differences in cannabis and prescribed pain medication use and found that 61.2% of the participants did experience a reduced need for pain medication as a result of using cannabis for their pain symptoms (29). Given the limitations of the study by Stillman et al., (29) which lacked information concerning the specific medication used and substituted, we designed the current study to include specific questions about pain medication used, as well as which medication was substituted with cannabis or cannabinoids. As a consequence, our findings suggest that opioids were the most substituted pain medication class. Given the abuse potential and side effect profile of these medications, subsequent research should investigate if cannabis or cannabinoids are better suited to manage pain symptoms in this population.

## 5. Limitations

The current study has some limitations. Firstly, although the survey was distributed across the country and included respondents from 40 states of the United States, most participants (36.1%, *n* = 82) were located in Florida. Secondly, most participants also identified as non-Hispanic, male, and Caucasian, which might not be the most accurate representation of the entire SCI population. Nevertheless, cannabis use has been found to be more prevalent in non-Hispanic males, as well as in states where medical and recreational use of cannabis has been legalized (43–45). Thirdly, with reference to neuropathic pain symptoms, we did not ask about participants' neuropathic pain intensity prior to starting to use cannabis but only focused on current pain intensity. Thus, it is impossible to evaluate the effectiveness of attempts in managing pain intensity using cannabis. However, since we were interested only in the perceived effects, we did include a question regarding participants' thoughts on whether cannabis has had any effect on neuropathic pain intensity, unpleasantness, interference, and dealing with the pain. In addition, our study did not include a control group comprising individuals with SCI and neuropathic pain who had not use cannabis or cannabinoids, making this group comparison not feasible. Finally, we also acknowledge that this study may highlight the positive effects of cannabis and cannabinoids on neuropathic pain intensity and associated outcomes by means of survey participant bias. Therefore, there may be an increased risk of bias favoring cannabis and cannabinoids over other commonly prescribed medications. That said, we also provided a comprehensive list of reported negative effects associated with cannabis and cannabinoid use (Figure 3F). We hope this presents a balanced approach when assessing the overall effectiveness of such compounds within people with neuropathic pain following SCI.

## 6. Conclusion

The current study adds to the pain management literature by suggesting that cannabis and cannabinoids might be beneficial in reducing SCI-associated neuropathic pain symptoms. Our findings also suggest that cannabis and cannabinoids are being used as substitutes for many prescribed pain medications. Although this evidence is preliminary, there is undoubtedly a strong need for large-scale placebo-controlled clinical trials examining the efficacy of cannabis and cannabinoids in reducing neuropathic pain and its influence in this population.

## Data Availability

The raw data supporting the conclusions of this article will be made available by the authors, without undue reservation.
