# Supplementary material for: A preliminary study evaluating self-reported effects of cannabis and cannabinoids on neuropathic pain and pain medication use in people with spinal cord injury
Source: Front Pain Res (Lausanne). 2023 Dec 21;4:1297223. doi: 10.3389/fpain.2023.1297223 (PMC10767995; doi:10.3389/fpain.2023.1297223)
Supplement: Supplementary file 1 [file Datasheet1.docx]

Supplementary Material

# Supplementary Data

Cannabis and Cannabinoids' Effect on Neuropathic Pain and Pain Medication Use in People with SCI

Start of Block: Block 1

Instructions PURPOSE OF THE STUDY:
We are interested in learning about how cannabis/cannabinoids affect neuropathic pain and medication use among people living with spinal cord injury (SCI). You will be asked to complete an online survey about your perspectives and experiences with cannabis/cannabinoids. This survey will take approximately 5 – 15 minutes, depending on your responses. *Please note that all of your responses will be kept confidential.

INFORMATION ABOUT NEUROPATHIC PAIN:
Neuropathic pain is a type of chronic pain that is located in an area at or below the level of the spinal cord injury. Neuropathic pain can be described as any of the following:
- Hot burning or painful cold
- Electric shock-like
- Pricking
- Pins and needles
- Sharp
- Stabbing
- Shooting
- Lacerating
- Squeezing
- Aching

INFORMATION ABOUT CANNABIS/CANNABINOIDS:
The cannabis plant produces compounds called cannabinoids, the two most common being CBD (cannabidiol) and THC (delta-9-tetrahydrocannabinol). THC is the psychoactive compound that causes euphoria and feelings of “high”, whereas CBD does not create a similar high sensation. Cannabis flowers contain both THC and CBD in different amounts based on the strain. Manufactured products can contain only CBD, since it can be completely free from THC. All products that contain THC, also contain some amount of CBD.

INSTRUCTIONS FOR PARTICIPATING:
Participation in this survey is voluntary, there are no negative consequences if you decide not to take the survey. If you decide to start the survey, you can stop at any time. Taking part in the survey has minimal to no risks, and there are no benefits or monetary compensation for participating.

To participate in this survey, you must have SCI and have experienced SCI-related neuropathic pain for at least 3 months. You must be over the age of 18 years and be able to understand the English language.

If you decide to participate in this survey, you will complete questions about some basic information, such as age and gender, details of your SCI and your day-to-day pain experiences as well as experiences with cannabis and/or cannabinoids. Your responses will not be identifiable.

If you have any questions or concerns regarding the survey or research, please feel free to contact the Principal Investigator Dr. Eva Widerström-Noga, ewiderstrom-noga@med.miami.edu.

If you have questions regarding your rights as a research participant, contact the University of Miami, Human Subject Research Office at hsro@miami.edu or (305)243-3195.

End of Block: Block 1

Start of Block: Questions

Consent By selecting “I agree” below, you consent to participate in this study.

- I agree (1)
- I do not agree (2)

Skip To: End of Survey If By selecting “I agree” below, you consent to participate in this study. = I do not agree

| Page Break |  |
| --- | --- |

Inclusion  Age Are you older than 18 years old?

- Yes (1)
- No (2)

Skip To: End of Survey If Are you older than 18 years old? = No

Inclusion SCI Do you have Spinal Cord Injury (SCI)?

- Yes (1)
- No (2)

Skip To: End of Survey If Do you have Spinal Cord Injury (SCI)? = No

Inclusion Pain Dur After your Spinal Cord Injury (SCI), have you experienced constant or intermittent pain for more than 3 months?

- Yes (1)
- No (2)

Skip To: End of Survey If After your Spinal Cord Injury (SCI), have you experienced constant or intermittent pain for more... = No

| 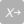 |
| --- |

Inclusion SCIPI Please read the following statements and select the response that applies to your pain:
STATEMENT 1:  My pain feels like electrical or electric shock like
 STATEMENT 2: My pain feels like pins and needles, or tingling
STATEMENT 3: The skin over the area of pain or inside my body where the pain is located feels hot or burning or cold or freezing
STATEMENT 4: My pain only occurs in an area of my body in which I have no feeling on the skin overlying that area

- One or none of the statement(s) is(are) true (1)
- Two or more of the statements are true (1)

Skip To: End of Survey If Please read the following statements and select the response that applies to your pain: STATEMENT... = One or none of the statement(s) is(are) true

Display This Question:

If By selecting “I agree” below, you consent to participate in this study. = I agree

Gender To which gender identity do you most identify?

- Male (1)
- Female (2)
- Transgender Male (3)
- Transgender Female (4)
- Gender Variant/Non-Conforming (5)
- Prefer not to answer (6)

Age What is your age?

- 18-30 (1)
- 31-45 (2)
- 46-60 (3)
- 61 or older (4)

State Which state do you live in?

▼ Alabama (7) ... Wyoming (57)

Ethnicity1 Are you of Hispanic, Latino, or Spanish origin?

- Yes (1)
- No (2)
- Prefer not to answer (3)

Ethnicity2 What racial group(s) do you identify as?

1. White (1)
2. Black or African American (2)
3. American Indian or Alaska Native (3)
4. Asian (4)
5. Native Hawaiian or Pacific Islander (5)
6. Prefer not to answer (6)
7. Other (Please specify): (7) __________________________________________________

Housing Please select your housing arrangement

- Living alone (2)
- Living with someone (1)
- Nursing home/facility (3)
- Homeless (4)
- Other (5) __________________________________________________

Education What is the highest degree or level of education you have completed?

- Less than a high school diploma (1)
- High school degree or equivalent (e.g. GED) (2)
- Some college, no degree (3)
- Associate degree (e.g. AA, AS) (4)
- Bachelor's degree (e.g. BA, BS) (5)
- Master's degree (e.g. MA, MS, MEd) (6)
- Doctorate or professional degree (e.g. MD, DDS, PhD) (7)

Marital Status What is your marital status?

- Single (never married) (1)
- Married, or in a domestic partnership (2)
- Widowed (3)
- Divorced (4)
- Separated (5)

Employment What is your current employment status?

1. Employed full time (40 or more hours per week) (1)
2. Employed part time (up to 39 hours per week) (2)
3. Unemployed (3)
4. Student (5)
5. Retired (6)
6. Self-employed (8)
7. Unable to work (9)

| Page Break |  |
| --- | --- |

SCI Level The Level of your Spinal Cord Injury

1. Cervical (1)
2. Thoracic (2)
3. Lumbar (3)
4. Unsure (Please Indicate): (4) __________________________________________________

SCI Grade The Grade of your Spinal Cord Injury

- Complete (No feeling or voluntary movement below the level of injury) (1)
- Incomplete (Some feeling and/or voluntary movement below the level of injury) (2)
- Unsure (Please explain): (3) __________________________________________________

How long after SCI How long has it been since your SCI?

- 6-12 months (1)
- 1-3 years (2)
- 4-6 years (3)
- 7-9 years (4)
- 10 or more years (5)

SCI Cause The cause(s) of your Spinal Cord Injury

1. Motor Vehicle Accident (MVA) - Pedestrian (1)
2. Motor Vehicle Accident (MVA) - Non-Pedestrian (2)
3. Violence (3)
4. Fall (4)
5. Sport-Related Injury (5)
6. Other (Please indicate): (6) __________________________________________________

Pain intensity On average, during the last week, how intense was your neuropathic pain on a scale from 0 to 10
 (0 no pain, 10 the most intense pain imaginable)?

|  | 0 | 1 | 2 | 3 | 4 | 5 | 6 | 7 | 8 | 9 | 10 |
| --- | --- | --- | --- | --- | --- | --- | --- | --- | --- | --- | --- |

| Intensity () | 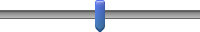 |
| --- | --- |

Pain unpleasantness On average, during the last week, how unpleasant was your neuropathic pain on a scale from 0 to 10
 (0 no unpleasantness, 10 the most unpleasant pain imaginable)?

|  | 0 | 1 | 2 | 3 | 4 | 5 | 6 | 7 | 8 | 9 | 10 |
| --- | --- | --- | --- | --- | --- | --- | --- | --- | --- | --- | --- |

| Unpleasantness () | 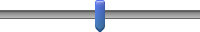 |
| --- | --- |

Interference A In general, how much has pain interfered with your day-to-day activities in the last week?
 (0 no interference, 10 extreme interference)?

|  | 0 | 1 | 2 | 3 | 4 | 5 | 6 | 7 | 8 | 9 | 10 |
| --- | --- | --- | --- | --- | --- | --- | --- | --- | --- | --- | --- |

| Interference () | 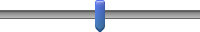 |
| --- | --- |

Interference M In general, how much has pain interfered with your overall mood in the last week?
 (0 no interference, 10 extreme interference)?

|  | 0 | 1 | 2 | 3 | 4 | 5 | 6 | 7 | 8 | 9 | 10 |
| --- | --- | --- | --- | --- | --- | --- | --- | --- | --- | --- | --- |

| Interference () | 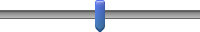 |
| --- | --- |

Interference S In general, how much has pain interfered with your ability to get a good night's sleep in the last week?
 (0 no interference, 10 extreme interference)?

|  | 0 | 1 | 2 | 3 | 4 | 5 | 6 | 7 | 8 | 9 | 10 |
| --- | --- | --- | --- | --- | --- | --- | --- | --- | --- | --- | --- |

| Interference () | 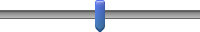 |
| --- | --- |

Deal with pain In general, how hard is it for you to deal with your pain?
 (0 no hard at all, 10 extreme hard)?

|  | 0 | 1 | 2 | 3 | 4 | 5 | 6 | 7 | 8 | 9 | 10 |
| --- | --- | --- | --- | --- | --- | --- | --- | --- | --- | --- | --- |

| Hard to deal () | 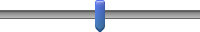 |
| --- | --- |

Medication Do you currently use any of the following medication?

1. Gabapentinoids (Gabapentin, Pregabalin) FusePaq Fanatrex, Gabarone, Gralise, Neurontin, Lyrica (1)
2. Serotonin and Norepinephrine reuptake inhibitors SSRIs (Duloxetine, Venlafaxine) Cymbalta, Yentreve,Vensir, Vencarm, Venlalix, Efexor, Venlablue (2)
3. Tricyclic Antidepressants (Nortriptyline, Amitriptyline ) Aventyl, Pamelor, Elavil, Vanatrip (3)
4. Topicals (5% Lidocaine, 8% Capsaicin) Anestacon, Burnamycin, Burn-O-Jel, Lida Mantle, Lidoderm, LidoRx, LMX4, LMX5, Qutenza (4)
5. Opioids (Tramadol, Tapentadol, Morphine, Oxycodone) Tramadol, OxyContin, Vicodin, Norco, Lortab, Percocet, MSiR, MS-Contin, Roxanol, RMS, Kadian and oramorph SR (5)
6. Neurotoxin (Botulinum toxin) (6)
7. Over-the-Counter Pain Medication (acetaminophen, aspirin, and nonsteroidal anti-inflammatory drugs (NSAIDs) such as ibuprofen, naproxen, and diclofenac) (16)
8. None (7)
9. Other (Please indicate): (8) __________________________________________________

| Page Break |  |
| --- | --- |

Cannabis Use ever Have you ever used Cannabis or Cannabinoids?

- Yes (1)
- No (2)

Skip To: End of Survey If Have you ever used Cannabis or Cannabinoids? = No

Cannabis use before Before the onset of your SCI, did you use cannabis/cannabinoids?

- Yes (1)
- No (2)

Cannabis Use after After the onset of your SCI, have you used cannabis/cannabinoids?

- Yes (1)
- No (2)

Cannabis Use current Do you currently use Cannabis/Cannabinoids?

- Yes (1)
- No (2)

Display This Question:

If Have you ever used Cannabis or Cannabinoids? = No

Cannabis no use If no, what is the reason you prefer not to use cannabis/cannabinoids?

1. It is Illegal (1)
2. I do not have a medical card (2)
3. It interferes with medication I currently take (3)
4. It is too expensive (4)
5. I am scared/nervous to try it (5)
6. Stigma/Stereotypes against Cannabis users (6)
7. It does not help with my symptoms (8)
8. It causes negative side effects (9)
9. Other (Please indicate): (7) __________________________________________________

| Page Break |  |
| --- | --- |

Display This Question:

If Do you currently use Cannabis/Cannabinoids? = Yes

Frequency How often do you use it?

- Less than monthly (1)
- Monthly (2)
- Weekly (3)
- Daily or almost daily (4)
- Multiple times a day (5)

Display This Question:

If Do you currently use Cannabis/Cannabinoids? = Yes

Frequency 2 On a day of use, How many times a day do you use Cannabis/Cannabinoids?

- Once a day (1)
- Twice a day (2)
- Three times a day (3)
- Four or more times a day (4)

Display This Question:

If Do you currently use Cannabis/Cannabinoids? = Yes

Time of the day What time of the day you prefer using Cannabis/Cannabinoids?

1. Morning (1)
2. During the day (2)
3. Evening (3)
4. No preference (5)

Display This Question:

If Do you currently use Cannabis/Cannabinoids? = Yes

Modality When using cannabis, do you usually:

1. Pipe (1)
2. Joint/Blunt (2)
3. Bong (3)
4. Vaporize (4)
5. Oil/Tincture (5)
6. Edible/Capsule/Food (6)
7. Topical (cream, lotion) (7)
8. Fresh Juice (8)
9. Other (Please indicate): (9) __________________________________________________

Display This Question:

If Do you currently use Cannabis/Cannabinoids? = Yes

Composition Which of the following do you consume? Select all that apply.

1. Cannabidiol (CBD) (1)
2. Delta-9-tetrahydrocannabinol (THC) (2)
3. I don't know (3)
4. Other (Please indicate): (4) __________________________________________________

Display This Question:

If Do you currently use Cannabis/Cannabinoids? = Yes

Amount How much cannabis do you use per week? 
(Multiply daily amount by 7)

- 1 gram or less (1)
- 2-4 grams (2)
- 5-7 grams (3)
- 8-10 grams (4)
- More than 10 grams (5)
- I don't know (6)
- Other (Please indicate): (7) __________________________________________________

Display This Question:

If Which of the following do you consume? Select all that apply. = Delta-9-tetrahydrocannabinol (THC)

Strain If you consume THC, which strain do you consume? Select all that apply.

1. Indica (Sedating) (1)
2. Sativa (Activating) (2)
3. Hybrid (3)
4. Not sure (4)

| Page Break |  |
| --- | --- |

Substitute for meds Do you, or have you ever used cannabis/cannabinoids as a substitute for pain medication?

- Yes (1)
- No (2)

Display This Question:

If Do you, or have you ever used cannabis/cannabinoids as a substitute for pain medication? = Yes

Substitute for meds2 If yes, which medication(s) do you substitute cannabis for?

1. Gabapentinoids (Gabapentin, Pregabalin) FusePaq Fanatrex, Gabarone, Gralise, Neurontin, Lyrica (1)
2. Serotonin and Norepinephrine reuptake inhibitors SSRIs (Duloxetine, Venlafaxine) Cymbalta, Yentreve,Vensir, Vencarm, Venlalix, Efexor, Venlablue (2)
3. Tricyclic Antidepressants (Nortriptyline, Amitriptyline ) Aventyl, Pamelor, Elavil, Vanatrip (3)
4. Topicals (5% Lidocaine, 8% Capsaicin) Anestacon, Burnamycin, Burn-O-Jel, Lida Mantle, Lidoderm, LidoRx, LMX4, LMX5, Qutenza (4)
5. Opioids (Tramadol, Tapentadol, Morphine, Oxycodone) Tramadol, OxyContin, Vicodin, Norco, Lortab, Percocet, MSiR, MS-Contin, Roxanol, RMS, Kadian and oramorph SR (5)
6. Neurotoxin (Botulinum toxin) (6)
7. Over-the-Counter Pain Medication (acetaminophen, aspirin, and nonsteroidal anti-inflammatory drugs (NSAIDs) such as ibuprofen, naproxen, and diclofenac) (8)
8. Other (Please indicate): (7) __________________________________________________

Help with pain Do Cannabis/Cannabinoids help with (or decrease) any of the following neuropathic pain related outcomes?

1. Pain Intensity (6)
2. Pain Unpleasantness (2)
3. Pain interference with day-to-day activities (3)
4. Pain interference with your overall mood (4)
5. Pain interference with your ability to get a good night's sleep (5)

Display This Question:

If Do Cannabis/Cannabinoids help with (or decrease) any of the following neuropathic pain related ou... = Pain Intensity

Pain intensity How much do cannabis/cannabinoids help with (or decrease) your neuropathic pain intensity?

- 25% or less (1)
- 30-50% (9)
- 51-75% (10)
- More than 75% (11)

Deal with pain How much do cannabis/cannabinoids help you to deal with your neuropathic pain?

- Not at all (1)
- A little (3)
- Some (4)
- A lot (5)

Positive effects What other effects have you experienced from Cannabis/Cannabinoids?
Select all that apply.

|  | Increase (1) | No effect (2) | Decrease (3) |
| --- | --- | --- | --- |
| Stress (1) |  |  |  |
| Anxiety/Depression (2) |  |  |  |
| Spasticity (4) |  |  |  |
| Insomnia (5) |  |  |  |
| Appetite (6) |  |  |  |
| Nausea/vomiting (7) |  |  |  |
| Focus/concentration (8) |  |  |  |
| Relaxation (9) |  |  |  |
| Other (Please Indicate): (11) |  |  |  |

Negative effects Have you experienced any of the following negative effects of Cannabis/cannabinoids? 
Select all that apply.

1. Dry mouth (1)
2. Cough (2)
3. Throat Irritation (3)
4. Dehydration (4)
5. Withdrawal symptoms (5)
6. Lightheadness/Dizziness (7)
7. Drowsiness/Fatigue (8)
8. Hallucinations/Delusions (9)
9. None (10)
10. Other (Please Indicate): (11) __________________________________________________

Benefit How beneficial has cannabis/cannabinoids been to your overall global wellbeing?

- Very much improved (1)
- Much improved (2)
- Minimally improved (3)
- No change (4)
- Minimally worse (5)
- Much worse (6)
- Very much worse (7)

End of Block: Questions

# Supplementary Figures


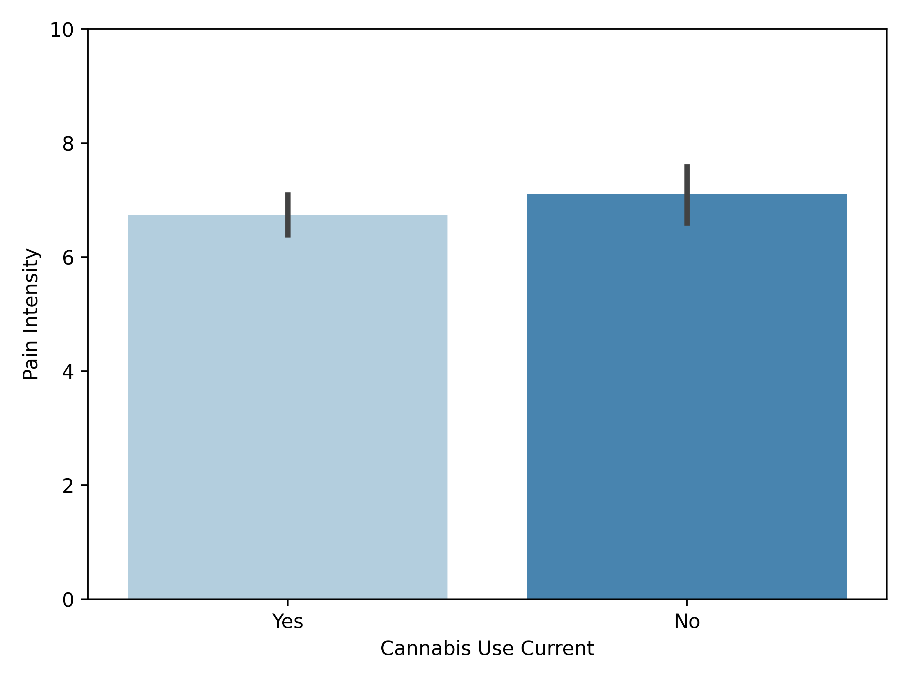


**Supplementary Figure 1.** Comparison of pain intensity in current vs. non-current users of cannabis/cannabinoids.


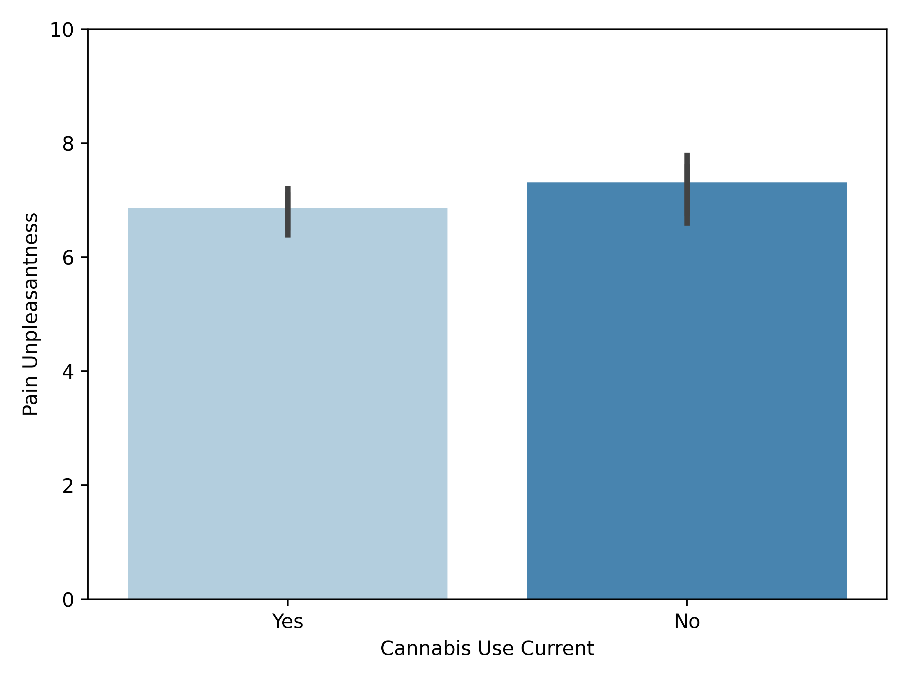


**Supplementary Figure 2.** Comparison of pain unpleasantness on current vs. non-current users of cannabis/cannabinoids.


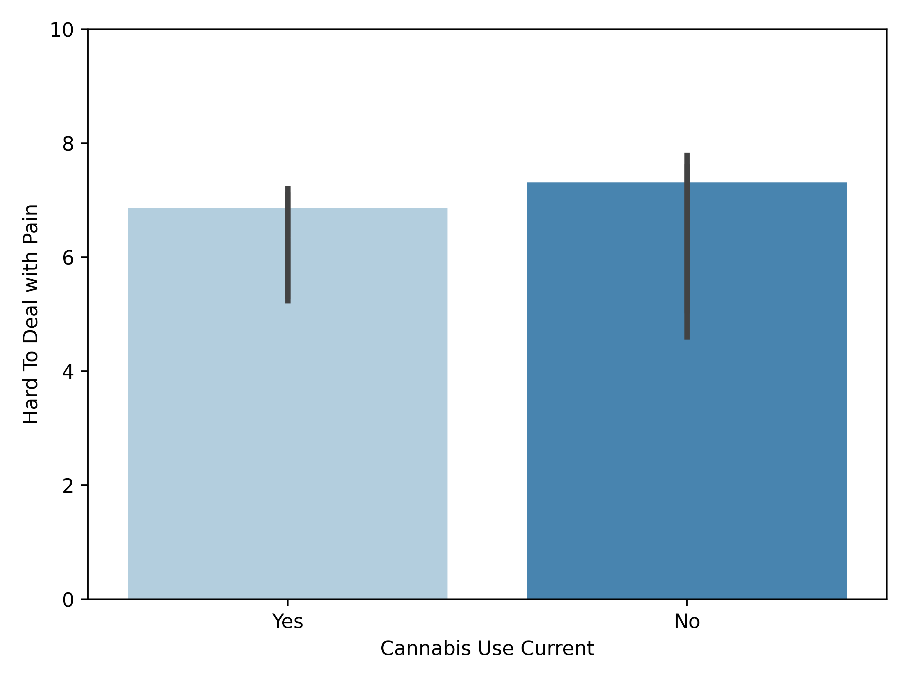


**Supplementary Figure 3.** Comparison of hard-to-deal-with pain on current vs. non-current users of cannabis/cannabinoids.


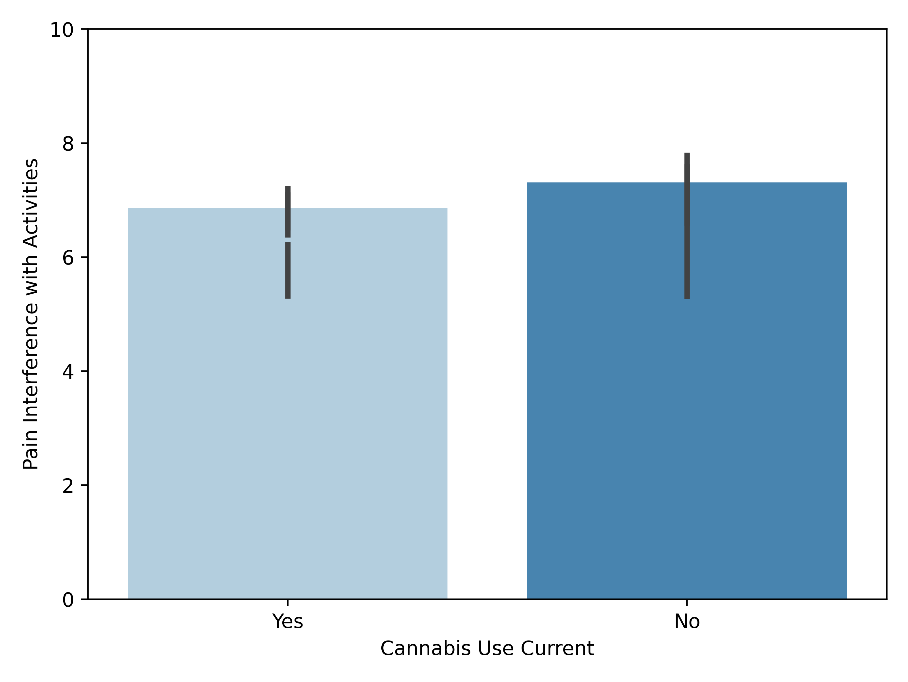


**Supplementary Figure 4.** Comparison of pain interference with activities on current vs. non-current users of cannabis/cannabinoids.


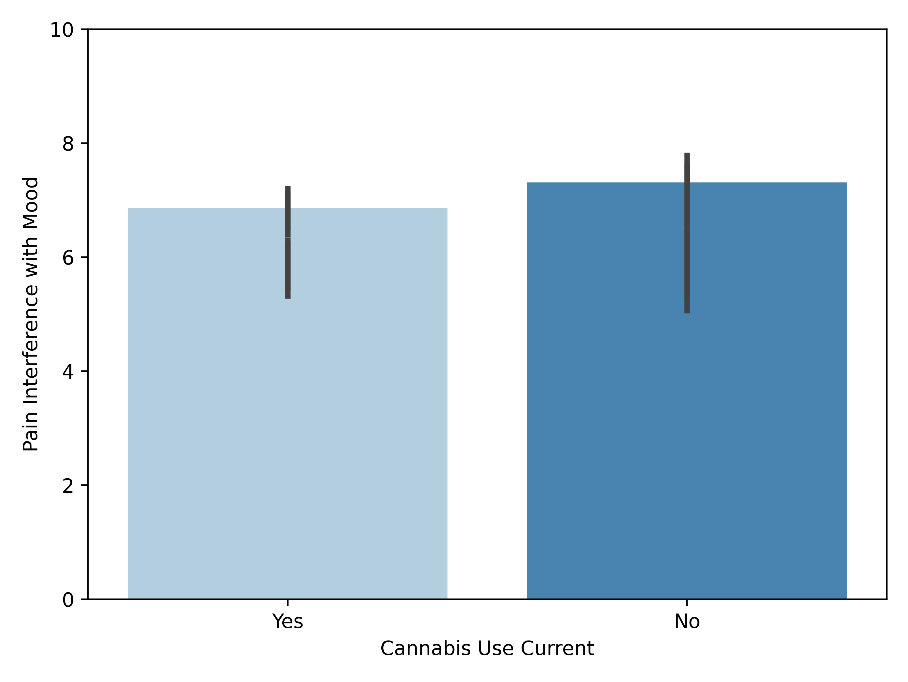


**Supplementary Figure 5.** Comparison of pain interference with mood on current vs. non-current users of cannabis/cannabinoids.


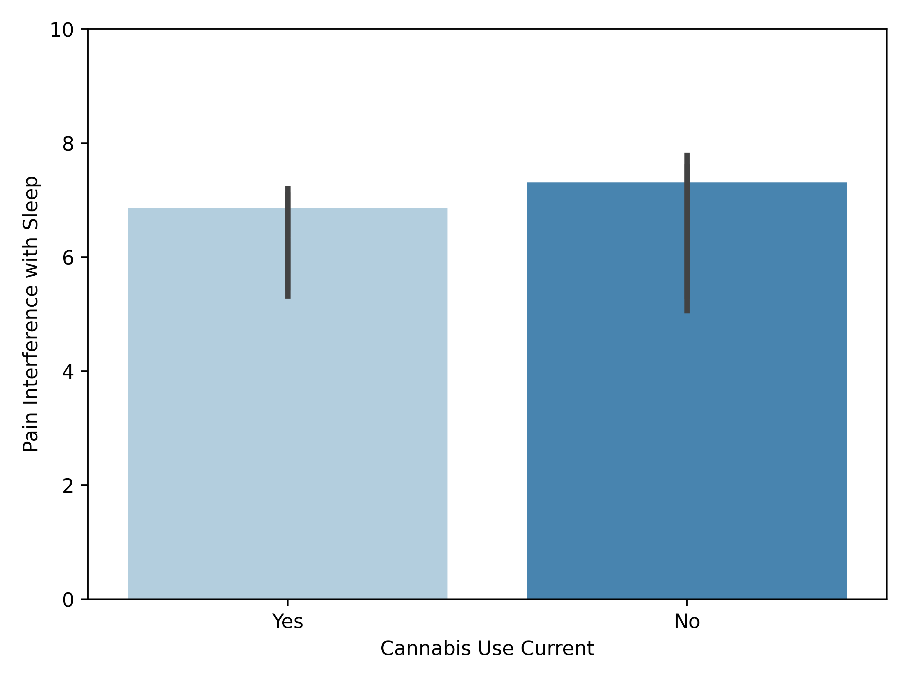


**Supplementary Figure 6.** Comparison of pain interference with sleep on current vs. non-current users of cannabis/cannabinoids.
